# Intestinal bacteria-derived tryptamine and its impact on human gut microbiota

**DOI:** 10.3389/frmbi.2024.1373335

**Published:** 2024-04-03

**Authors:** Nize Otaru, Anna Greppi, Serafina Plüss, Janina Zünd, Denisa Mujezinovic, Jana Baur, Ekaterina Koleva, Christophe Lacroix, Benoit Pugin

**Affiliations:** ^1^ Laboratory of Food Biotechnology, Department of Health Sciences and Technology, ETH Zürich, Zürich, Switzerland; ^2^ Nutrition Research Unit, University Children’s Hospital Zürich, Zürich, Switzerland

**Keywords:** tryptamine, tryptophan decarboxylase, acid stress, gut microbes, human gut microbiota, community composition, carbohydrate metabolism, growth inhibition

## Abstract

Tryptamine, a neuromodulator derived from tryptophan, has been shown to significantly impact the host gut homeostasis through its production by the gut microbiota. However, the characterization of tryptamine-producing gut bacteria remains limited, the factors regulating tryptamine production largely unexplored, and its effects on the rest of the gut microbial community unknown. In this study, we screened 13 intestinal strains closely related to known tryptamine producers, characterized their production kinetics, and evaluated whether tryptophan decarboxylation to tryptamine contributes to acid stress tolerance, as shown in other amino acid-dependent acid tolerance systems. We also examined the impact of tryptamine on the composition and function of four healthy human gut microbiota by conducting 48-h *ex vivo* fecal batch fermentations. To complement the *ex vivo* experiments, we tested the effect of tryptamine exposure (range: 0.5–8 mM) on the growth of 18 intestinal strains. We identified tryptamine production in five taxa, i.e., *Enterocloster asparagiformis*, *Blautia hansenii*, *Clostridium nexile*, *Clostridium sporogenes*, and *Ruminococcus gnavus*, with *R. gnavus* DSM 108212 accumulating up to 3.4 mM tryptamine after 48 h. An increased tryptophan concentration led to higher tryptamine production. However, tryptamine production was not promoted at low pH and may not protect cells from acid-induced cellular damage. Exposing gut microbial communities to 2.4 mM tryptamine caused mild changes in gut microbiota function and composition. All donors showed reduced carbohydrate consumption after 5 h, leading to donor-specific alterations of short-chain fatty acids (SCFAs) (i.e., propionate, acetate, butyrate) and branched-chain fatty acids (BCFAs) (i.e., isobutyrate and isovalerate) after 48 h. Tryptamine also induced a mild change of community structure, with a consistent reduction in the phylum *Bacteroidota* as well as amplicon sequence variants (ASVs) related to the genera *Bacteroides*, *Blautia*, and *Faecalibacterium*. We confirmed the sensitivity of *Bacteroides* and *Faecalibacterium* strains *in vitro* at concentrations of 2 mM and above. Multiple gut commensals remained unaffected when exposed to 8 mM tryptamine. Taken together, our findings demonstrated that intestinal bacteria-derived tryptamine is a bioactive molecule that not only alters host homeostasis locally but also modulates the physiology of gut microbial communities. The specific mechanism through which tryptamine exerts its inhibitory effects on specific gut microbes while leaving others unaffected remains to be elucidated.

## 1 Introduction

The human gastrointestinal tract is a complex and dynamic ecosystem inhabited by a diverse community of microorganisms known as the gut microbiota. The gut microbiota plays a significant role in modulating human health (Fung et al., 2017; Fan and Pedersen, 2021), in part through the production of bioactive molecules (Krautkramer et al., 2021) such as biogenic amines (Pugin et al., 2017). Biogenic amines are small amino acid-derived molecules with diverse biological activities, including hormonal, immune, neuromodulatory, and neurotransmitter functions (Erdag et al., 2018). Among these, tryptamine (Try), a potent neuromodulator (Khan and Nawaz, 2016), is produced through the catabolism of tryptophan (Trp) by the enzyme tryptophan decarboxylase (also known as aromatic L-amino acid decarboxylase) (Williams et al., 2014).

Although tryptophan decarboxylase is rare in bacteria, a previous metagenomic study revealed that at least 10% of the human population harbors gut bacteria encoding this enzyme (Williams et al., 2014). The tryptophan levels in healthy human feces, influenced by diet (Keszthelyi et al., 2009), range from micro- to millimolar concentrations, while tryptamine is found in the micromolar range (Nikolaus et al., 2017; Dong et al., 2020; Desmons et al., 2022; Zhai et al., 2023). Once released, bacteria-derived tryptamine can modulate host homeostasis by interacting with 5-HT_4_R receptors on colonocytes, leading to increased gastrointestinal motility, fluid secretion (Williams et al., 2014; Bhattarai et al., 2018), and mucus release (Bhattarai et al., 2020). Bacteria-derived Try also attenuates weight loss, colitis severity, and barrier disruption in dextran sulfate sodium-induced colitis mouse model (Bhattarai et al., 2020). Moreover, tryptamine has been found to weaken the production of pro-inflammatory cytokines in cultured macrophages through the activation of AhR receptors (Krishnan et al., 2018) and suppress neuroinflammation in a murine model of multiple sclerosis (Dopkins et al., 2021). In the latter study, Try treatment also led to an alteration of the gut microbiota, with a decrease in *Bacteroides* relative abundance and an increase in cecal butyrate levels (Dopkins et al., 2021), suggesting a direct impact of Try on the gut microbial community.

Despite the ample evidence highlighting the interaction between gut Try and the host, the mechanisms underlying bacteria-derived Try production and regulation within the gut remain poorly understood. A limited number of Try-producing strains have been confirmed *in vitro* (Williams et al., 2014), including *Ruminococcus gnavus* ATCC 29149 and *Clostridium sporogenes* ATCC 155797, and the exact role and beneficial function of tryptophan decarboxylase for bacterial cells remain largely unknown. It is plausible that tryptophan decarboxylase may contribute to a mechanism of acid stress resistance, akin to other bacterial amino acid decarboxylases (Guan and Liu, 2020), by consuming excessive cytoplasmic protons (H^+^) during tryptophan conversion.

In this study, we aimed at further characterizing Try production and regulation in gut commensals. We screened a panel of bacterial intestinal strains closely related to known tryptamine producers, evaluated the kinetics of Try production, and assessed the regulation of Try in response to acid stress. Furthermore, we examined how Try impacts the composition and function of adults’ and toddlers’ gut microbiota *ex vivo* and determined Try’s sensitivity of specific intestinal strains using pure culture assays.

## 2 Materials and methods

### 2.1 Bacterial strains and culture conditions

A total of 26 bacterial strains were used in this study (**Supplementary Table 1**). They were acquired from the German Collection of Microorganisms and Cell Culture GmbH (DSMZ, Braunschweig, Germany) and the American Type Culture Collection (ATCC, Manassas, Virginia, USA) and obtained from our own collection. The strains were stored at −80°C in 25% (v/v) anaerobic glycerol stocks and routinely cultivated at 37°C, without shaking, in Hungate tubes containing anaerobic modified yeast extract casitone and fatty acid medium (mYCFA; O_2_-free CO_2_ head gas) consisting of (per liter): 10 g Amicase (Sigma-Aldrich, St. Louis, MO, USA), 2.5 g yeast extract (Sigma-Aldrich), 1.5 g meat extract (Sigma-Aldrich), 4 g NaHCO_3_, 0.45 g K_2_HPO_4_, 0.45 g KH_2_PO_4_, 0.9 g NaCl, 0.9 g (NH_4_)_2_SO_4_, 90 mg MgSO_4_, 90 mg CaCl_2_, 10 mg hemin, 1 g L-cysteine·HCl, 10 μg biotin, 10 μg cobalamin, 30 μg p-aminobenzoic acid, 50 μg folic acid, 150 μg pyridoxamine, and 5.75 mL of volatile fatty acid solution (Bircher et al., 2018). Except when otherwise stated, the medium was supplemented with glucose, cellobiose, and soluble starch (2 g/L each) as carbon sources, and the pH was adjusted to 6.5. When appropriate, mYCFA was also supplemented with L-tryptophan (Trp; from 5 to 24.5 mM). The procedures for anaerobic medium preparation in Hungate tubes were performed as previously described (Bircher et al., 2018).

### 2.2 *In vitro* screening for tryptamine producers

A total of 13 strains (**Supplementary Table 1**), closely related to known tryptamine producers or previously identified *in silico* as potential tryptamine producers (Williams et al., 2014), were reactivated from glycerol stocks by a 1.25% (v/v) inoculation of anaerobic mYCFA. Pre-cultures were incubated for 24 h at 37°C, and screening was initiated by inoculating 1.25% (v/v) of pre-cultures in 8 mL of fresh mYCFA containing 24.5 mM Trp. After 24 and 48 h of incubation at 37°C, 1 mL of bacterial cultures was centrifuged (14,000 × *g*, 10 min, 4°C), and the supernatants were directly processed for tryptamine (Try) quantification (described below). Each strain was screened using three independent replicates.

### 2.3 Growth kinetics and tryptamine production

To identify growth phases associated with Try production, *R. gnavus* DSM 108212, *B. hansenii* DSM 20583, *C. sporogenes* DSM 795, and *C. nexile* DSM 1787 were reactivated in mYCFA (1.25%, v/v) for 24 h at 37°C. The pre-cultures were subsequently transferred (1.25%, v/v) into mYCFA, grown to an OD_600_ of ~0.3, and re-inoculated (1.25%, v/v) into mYCFA containing 15 mM tryptophan to initiate the kinetic analysis. OD_600_ and extracellular Try concentrations (supernatants: 14,000 × *g*, 10 min, 4°C) were monitored throughout 48 h of growth at 37°C. Each strain was evaluated using three independent replicates.

### 2.4 Impact of tryptophan concentration on tryptamine production

To investigate the impact of Trp concentration on Try production, *R. gnavus* DSM 108212, *B. hansenii* DSM 20583, and *C. sporogenes* DSM 795 were reactivated in mYCFA (1.25%, v/v) for 24 h at 37°C. The analysis was then initiated by transferring the pre-cultures (1.25%, v/v) into mYCFA containing 10 or 24 mM Trp at pH 6.5. OD_600_ and extracellular Try concentrations (supernatants: 14,000 × *g*, 10 min, 4°C) were monitored after 6, 24, and 48 h of growth at 37°C. Each strain was evaluated using three independent replicates.

### 2.5 Acid stress response and cell viability

The effect of mild acid stress on the induction of Try production was first evaluated with actively growing cells of *R. gnavu*s ATCC 29149. The cells were reactivated in mYCFA (1.25%, v/v) for 24 h at 37°C. The pre-cultures were then transferred (1.25%, v/v) into mYCFA containing 5 mM Trp and grown to an OD_600_ of ~0.2, after which the pH was reduced to pH 5.5 and 5.0 using 2.5 M HCl anaerobic solution. The cells were incubated for a total of 24 h at 37°C, and the Try concentration in supernatants (14,000 × *g*, 10 min, 4°C) was evaluated 2 and 18 h after stress induction. Each condition was tested in three independent replicates.

Subsequently, the effect of acid stress on Try production and survival was tested in resting cells of *R. gnavus* DSM 108212 and *C. sporogenes* DSM 795, as previously described (Otaru et al., 2021). Briefly, after reactivation in mYCFA (1.25%, v/v) for 24 h at 37°C, the cells were transferred (1.25%, v/v) into mYCFA and grown for 15 h to reach the late exponential phase (OD_600_ ~1.0). In an anaerobic chamber (10% CO_2_, 5% H_2_, and 85% N_2_, Coy Laboratory Products, Inc., Ann Arbor, MI, USA), 1-mL bacterial cultures were harvested, centrifuged (6000 × *g*, 8 min, room temperature), and washed with sterile 0.9% NaCl solution. Acid stress was induced by resuspending the cell pellets in 1 mL carbohydrate-depleted mYCFA with or without 5 mM Trp at three different pH conditions each, i.e., 6.7, 5.4, and 4.3, followed by anaerobic incubation at 37°C. Cell viability, as well as Try and Trp concentrations (supernatants: 14,000 × *g*, 10 min, 4°C), was measured after 1 h of incubation. Each condition was tested in three independent replicates.

Cell viability was determined as described before (Otaru et al., 2021), by membrane integrity analysis using flow cytometry with two different dyes, i.e., the cell-impermeable molecule propidium iodide (PI, Life Technologies, Zug, Switzerland) and the cell-permeable SYBR Green I stain (SG, Life Technologies). Briefly, the bacterial cultures were diluted with phosphate-buffered saline (PBS) solution to achieve an approximate concentration of 10^7^ cells/mL. The resulting dilutions (30 μL) were then mixed with 267 μL PBS and 3 μL SG (1× concentration) to assess the total cell counts or with 3 μL SGPI solution (SG: 1× concentration; PI: 4 μM) to assess the intact cells. The mixtures were incubated for 30 min at room temperature in the dark. Samples were analyzed with a Cytomics FC 500 (Beckman Coulter GmbH, Krefeld, Germany) equipped with an air-cooled argon ion laser emitting 20 mW at 488 nm. Data analysis was performed with Kaluza Analysis 2.1 (Beckman Coulter GmbH). Total (SG-stained samples) or intact cells (SGPI-stained samples) were selected using the green and red detection channels. Cell viability (%) was calculated as the ratio of intact to total cells.

### 2.6 Impact of tryptamine on adult and toddler gut microbiota *ex vivo*

Fresh fecal samples from two healthy toddlers (toddler #1: female, 14-month-old; toddler #2: male, 17-month-old) and two healthy adults (adult #1: male, 34-year-old; adult #2: female, 23-year-old) were used to conduct fecal-derived *ex vivo* batch fermentations. Within an anaerobic chamber (10% CO_2_, 5% H_2_, and 85% N_2_; Coy Laboratory Products, Inc.), fecal slurries were prepared by mixing ~2 g feces, 10 autoclaved glass beads (borosilicate, diameter: 3 mm) and 20 mL anaerobic peptone water (Oxoid™, Thermo Scientific, Waltham, USA), and the suspensions were vortexed for 2 min. After the sedimentation of fecal particles, the upper layers were transferred into Hungate tubes which were tightly closed for use outside the anaerobic chamber. Balch-type tubes containing 20 mL toddler-mYCFA (per liter: 3 g lactose, 0.3 g xylan, 0.3 g arabinogalactan, 0.3 g starch, and 2 g mucin as added carbon sources in mYCFA) or adult-mYCFA (per liter: 1 g cellobiose, 1 g xylan, 1 g arabinogalactan, 1 g starch, and 1 g mucin as added carbon sources in mYCFA) were anaerobically spiked with 2 mL of 0.1 M HCl solution (control) or 2 mL of tryptamine (stock at 5.9 mg/mL tryptamine in 0.1 M HCl; final concentration: 2.4 mM tryptamine). Finally, 0.2 mL of fecal slurry was inoculated in the corresponding media to initiate the fermentation, and the tubes were incubated at 37°C for 48 h. Samples (2 mL) were harvested anaerobically using sterile syringes after 0, 5, 24, and 48 h and centrifuged at 14,000 × *g* for 10 min at 4°C to separate cell pellets (for 16S rRNA community analysis) and supernatants (for metabolite quantification and pH), and the resulting samples were stored at -80°C until further analysis.

### 2.7 16S rRNA metabarcoding analysis

DNA was isolated from the cell pellets obtained from 2 mL of batch fermentation samples using the FastDNA Spin Kit for Soil (MP Biomedicals, Illkirch-Graffenstaden, France), following the manufacturer’s instructions. The bacterial community was analyzed using 16S rRNA gene sequencing. Briefly, a two-step PCR approach was used to target the V4 region using primers 515F (5′-GTGCCAGCMGCCGCGGTAA-3′) and 806R (5′-GGACTACHVGGGTWTCTAAT-3′) (Microsynth AG, Balgach, Switzerland). The samples were barcoded using Nextera XT v2 indexes (Illumina, San Diego, CA, USA) and pooled in equimolar concentration. Sequencing was performed with the Illumina MiSeq platform (Genetic Diversity Centre, ETH Zurich, Switzerland) using v2 chemistry supplemented with PhiX 20 pM (10%) and 250 × 2 read length. Raw data were processed using the DADA2 R package (v1.14.1) to obtain exact amplicon sequence variants (ASVs) using the metabaRpipe R package (Constancias and Mahé, 2022) as previously described (Kundra et al., 2022). Taxonomy was assigned to ASVs using DADA2 against SILVA database (v138.1). Determination of the phylogenetic relatedness of ASVs and alpha diversity (observed and Shannon indexes) and beta diversity analyses (weighted and unweighted UniFrac) were performed using the phyloseq (McMurdie and Holmes, 2013) and the DivComAnalyses packages (Constancias and Sneha-Sundar, 2022), and differential abundance was established using DESeq2 (Love et al., 2014).

### 2.8 Metabolite quantification

The concentrations of Try and Trp in bacterial supernatants were determined by ultra-performance liquid chromatography equipped with a diode array detector (UPLC-DAD) and pre-column derivatization with diethyl ethoxymethylenemalonate (DEEMM), as described before (Otaru et al., 2021). Briefly, the derivatization was performed by mixing 200 μL of supernatant sample with 350 μL of borate buffer (1 M H_3_BO_3_ adjusted to pH 9 with NaOH), 150 μL methanol, 8 μL of internal standard (2 g/l L-2-aminoadipic acid, Sigma-Aldrich), and 7 μL DEEMM (VWR International GmbH). The reaction mix was incubated at room temperature in an ultrasound bath for 45 min and was subsequently heated at 70°C for 2 h to stop the derivatization. The samples were passed through a 0.2-μm nylon membrane filter and stored at 4°C until UPLC-DAD analysis. An ACQUITY UPLC H-Class system (Waters Corp., Milford, MA, USA) coupled to a diode array detector at 280 nm was used to detect the derivatized molecules. The separation (1-µL injection per sample) was performed at 40°C using an ACQUITY BEH C18 VanGuard pre-column (1.7-μm particle size, 2.1 mm × 5 mm; Waters Corp.) connected to an ACQUITY BEH C18 column (1.7-μm particle size; 2.1 × 100 mm; Waters Corp.), a flow rate of 0.46 mL/min, and a gradient consisting of (A) 25 mM acetate buffer (pH 6.6), (B) 100% methanol, and (C) 100% acetonitrile [gradient described in detail in Otaru et al. (2021)]. The detection limit for Try and Trp was 10 µM. The data were processed using Empower 2 software (Waters Corp.).

Carbohydrates (glucose, galactose, and lactose), intermediate metabolites (formate, succinate, and lactate), short-chain fatty acids (SCFAs; acetate, butyrate, and propionate), and branched-chain fatty acids (BCFAs; isobutyrate and isovalerate) were quantified by high-performance liquid chromatography equipped with a refractive index detector (HPLC-RI) as previously described (Poeker et al., 2018). Briefly, 200 μL of bacterial supernatant was passed through a 0.2-μm nylon membrane filter prior to HPLC-RI analysis. The separation was carried out with a LaChrom HPLC-System (Merck-Hitachi, Tokyo, Japan) using a SecurityGuard Cartridges Carbo-H (4 × 3.0 mm; Phenomenex Inc., Torrance, CA, USA) connected to a Rezex ROA-Organic Acid H+ (300 × 7.8 mm; Phenomenex Inc.) column. The samples (40-μL injection) were eluted at 40°C under isocratic conditions (10 mM H_2_SO_4_ and flow rate 0.4 mL/min), and the analytes were quantified using a refractive index detector L-2490 (Merck Hitachi). Data were processed using EZChrom software (Agilent, Santa Clara, CA, USA).

### 2.9 Determination of tryptamine sensitivity across individual intestinal strains

To evaluate the effect of Try on the growth of 18 intestinal strains (**Supplementary Table 1**), all strains were reactivated (1.25%, v/v) into anaerobic mYCFA until saturation (approximately ~20 h) at 37°C and were then diluted to OD_600_ 0.3 with mYCFA. The assay was then initiated in the anaerobic chamber by inoculating (1%, v/v) the diluted bacterial suspensions into 96-well plates (SPL Life Sciences Co. Ltd., Gyeonggi-do, South Korea) containing 200 µL mYCFA with incremental concentrations of tryptamine (from 0.5 to 8 mM). All plates also contained mYCFA without Try (control wells; unperturbed growth). The preparation of the medium for growth in the multiwell setup in the anaerobic chamber was based on the method previously described (Zünd et al., 2024). The plates were incubated at 37°C in a Tecan Infinite M200 PRO plate reader (Tecan Group Ltd., Männedorf, Switzerland), and OD_600_ was monitored for 24 h. Subsequently, all wells were re-inoculated (1%, v/v) into 96-well plates containing fresh mYCFA without tryptamine. These plates were incubated at 37°C, and OD_600_ was monitored for an additional 24 h. To quantify growth, we calculated the area under the curve (AUC) from OD_600_ measurements using the trapezoidal rule. To determine growth with Try as compared to the control (% growth), AUCs were normalized by dividing the mean AUC for specific Try concentrations by the mean AUC of unperturbed growth (control) of the same strain within the same plate. Each condition was tested in two independent replicates.

### 2.10 Data analysis and statistics

Data were analyzed and visualized using GraphPad Prism (v9.2.0) or R (v3.6.3) software. Statistical analysis was performed by multiple unpaired *t*-test, including Holm–Šídák test to correct for multiple comparison. The significance level was set to *p* < 0.05. The effects of Try treatment on microbiota alpha and beta diversity were tested using unpaired *t*-test (normal distribution assessed using the Shapiro–Wilk test) and PERMANOVA analysis, respectively, using the phyloseq R package (McMurdie and Holmes, 2013).

## 3 Results

### 3.1 Screening and characterization of tryptamine production by intestinal strains

**Figure 1:**
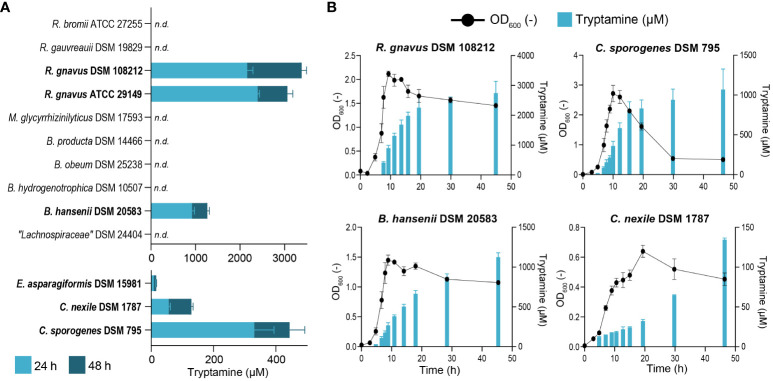
Characterization of tryptamine-producing strains. **(A)** Screening for tryptamine production after 24 and 48 h in the supernatant of 13 intestinal strains grown at 37°C in mYCFA containing 24.5 mM tryptophan. Tryptamine producers are highlighted in bold. Data are mean and standard deviation from three biological replicates. n.d., not detected. Tryptamine detection limit: 10 µM. **(B)** Growth (OD_600_) and tryptamine production kinetics by *R. gnavus* DSM 108212, *C. sporogenes* DSM 795, *B. hansenii* DSM 20583, and *C. nexile* DSM 1787 grown at 37°C for 48 h in mYCFA containing 15 mM tryptophan. Data are mean and standard deviation of three independent replicates.

To identify tryptamine-producing strains *in vitro*, we first screened 13 intestinal strains closely related to known Try producers (i.e., *Ruminococcus gnavus* ATCC 29149 and *Clostridium sporogenes* ATCC 155797) or previously identified *in silico* as potential Try producers (i.e., *Blautia hansenii*, *Clostridium nexile*, and *Enterocloster asparagiforme*) (Williams et al., 2014). We evaluated extracellular Try production after 24 and 48 h of growth in mYCFA containing 24.5 mM Trp. This elevated concentration was chosen to enhance the detectability of tryptamine using our analytic method by amplifying the metabolic capabilities of the tested strains. Six strains produced tryptamine, namely, *C. sporogenes* DSM 795 (Clostridiaceae family), *C. nexile* DSM 1787, *E. asparagiformis* DSM 15981, *B. hansenii* DSM 20583, *R. gnavus* DSM 108212, and the positive control *R. gnavus* ATCC 29149 (Lachnospiraceae family) (**Figure 1A**). No tryptamine was detected in *Lachnospiraceae bacterium* DSM 24404, *Blautia hydrogenotrophica* DSM 10507, *Blautia obeum* DSM 25238, *Blautia producta* DSM 14466, *Mediterraneibacter glycyrrhizinilyticus* DSM 17593 (Lachnospiraceae family), *Ruminococcus gauvreauii* DSM 19829, and *Ruminococcus bromii* ATCC 27255 (Oscillospiraceae family) under the tested conditions (limit of detection: 10 µM; **Figure 1A**). The lowest tryptamine concentration was detected in *E. asparagiformis* DSM 15981 (16 ± 1 µM after 48 h) and the highest in *R. gnavu*s DSM 108212 (3883 ± 110 µM after 48 h; **Figure 1A**). Next, we evaluated whether Try production was associated with a particular bacterial growth phase. Each newly identified tryptamine-producing strain was grown in mYCFA containing 15 mM Trp (*E. asparagiformis* DSM 15981 was excluded because the quantified Try was close to the limit of detection of the method), and OD_600_ and extracellular Try levels were monitored during 48 h (**Figure 1B**). In all four tested strains, Try formation was mixed-growth-associated (Sakthiselvan et al., 2019), with initial detection at low levels during the exponential growth phase (before 5 h growth), and additional production during the stationary phase to reach the highest levels after 48 h (ranging from 134 ± 2 µM for *C. nexile* DSM 1787 and up to 2,746 ± 325 µM for *R. gnavus* DSM 108212; **Figure 1B**). Finally, we assessed whether the concentration of available tryptophan could affect tryptamine production. Increasing Trp concentration in the media from 10 to 24 mM resulted in a significant increase of Try production in *R. gnavus* DSM 108212 (+ 2.7 mM max. after 24 h), *B. hansenii* DSM 20583 (+ 1.2 mM max. after 24 h), and *C. sporogenes* DSM 795 (+ 0.5 mM max. after 48 h), with no major impact on the growth (**Supplementary Figure 1**).

### 3.2 Acid stress may not induce tryptamine production in intestinal strains

Because the decarboxylation of amino acids into biogenic amines often constitutes a mechanism of acid stress tolerance in bacteria (Guan and Liu, 2020), we next investigated if tryptamine production was modulated by pH and could potentially be induced at low pH to protect cells against acid stress-mediated damage.

First, we investigated if higher Try levels were produced when applying acid stress to an equivalent amount of actively growing cells. *R. gnavus* ATCC 29149 was grown at 37°C until OD_600_ ~0.2 in mYCFA (with 5 mM Trp), after which the pH was reduced to 5.0 and 5.5 (from 6.2; control), and Try concentration was quantified 2 and 18 h after the acid stress. Compared to unperturbed control cells, the Try concentration normalized per OD_600_ did not significantly increase post-stress, yet slightly higher but non-significant normalized Try levels were observed at pH 5.0 compared to the control (6,293 ± 203 μM/OD_600_ vs. 5,333 ± 421 μM/OD_600_; *p* = 0.07) at 18 h post-stress (**Supplementary Figure 2**).

**Figure 2:**
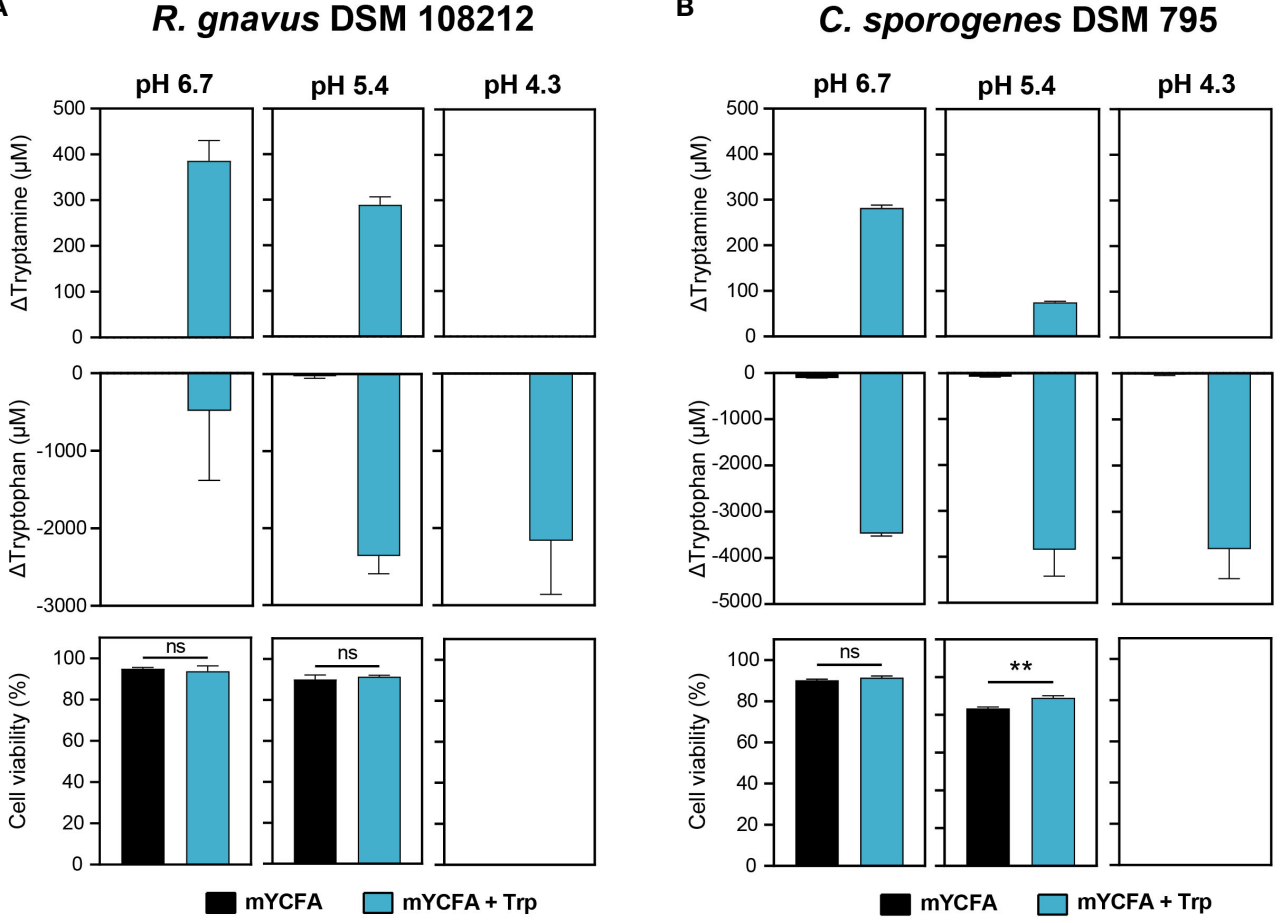
Effect of acid stress on tryptamine and tryptophan concentrations and cell viability in **(A)** *R. gnavus* DSM 108212 and **(B)** *C. sporogenes* DSM 795. The cells were exposed to mYCFA (black) or mYCFA containing 5 mM tryptophan (blue) at pH 6.7, 5.4, or 4.3 for 1 h at 37°C. Data are mean and standard deviation of three independent replicates. Significances were calculated by using unpaired *t*-test. **p < 0.01; ns, not significant (*p* > 0.05).

To further examine Try production in response to acid stress and assess if Try production was associated with increased cell viability under acidic conditions, *R. gnavus* DSM 108212 and *C. sporogenes* DSM 795 were grown in mYCFA until the late exponential growth phase (OD_600_ ~1.0). Subsequently, they were exposed for 1 h to carbohydrate-depleted mYCFA at three different pH values (i.e., 6.7, 5.4, and 4.3), with and without the precursor Trp (5 mM). In the absence of Trp, tryptamine was not produced under any of the tested pH conditions (**Figure 2**). When Trp was supplemented, maximum Try production was detected at the highest pH of 6.7 (386 ± 36 µM for *R. gnavus* DSM 108212 and 282 ± 5 µM for *C. sporogenes* DSM 795), decreased at pH 5.4 (289 ± 14 µM for *R. gnavus* DSM 108212 and 75 ± 2 µM for *C. sporogenes* DSM 795), and was not detected at pH 4.3 in both strains (**Figure 2**). When normalized per number of intact cells, the Try produced remains highest at pH 6.7 *versus* pH 5.4 (3.2 nM/cell vs. 1.7 nM/cell for *R. gnavus* and 1.9 nM/cell vs. 0.5 nM/cell for *C. sporogenes*). On the other hand, Trp was consumed by both strains at all pH levels, including at pH 4.3 where no tryptamine was detected, with 2,164 ± 568 µM and 3,807 ± 522 µM Trp consumed by *R. gnavus* DSM 108212 and C*. sporogenes* DSM 795, respectively (**Figure 2**). No difference of cell viability was observed in response to Try production in *R. gnavus* DSM 108212 at pH 6.7 and 5.4 (**Figure 2A**). In *C. sporogenes* DSM 795, no difference of cell viability was observed at pH 6.7, but a slight increase of viable cells was observed at pH 5.4 in the presence of Trp (89.1% vs. 83.5%, *p* = 0.002; **Figure 2B**). For both strains, no viable cells were detected after 1 h of exposure to pH 4.3 in the presence or absence of Trp (**Figure 2**).

Our data altogether suggest that tryptamine production may not be induced by acid stress in *R. gnavus* and *C. sporogenes* under the tested conditions and therefore may not constitute an effective mechanism of protection against acid stress.

### 3.3 Tryptamine exhibits limited effects on human gut microbiota function and structure *ex vivo*

Previous studies showed a significant modulatory effect of bacteria-derived tryptamine on intestinal homeostasis (Williams et al., 2014; Bhattarai et al., 2018), with indications of a potential direct impact of Try on the gut microbiota function and composition (Dopkins et al., 2021). To evaluate this interaction, we performed *ex vivo* fecal batch fermentation and exposed four healthy gut microbiota (two adults and two toddlers) to 2.4 mM tryptamine. We then assessed how Try could influence the metabolism and composition of gut microbial communities throughout the fermentation period (i.e., 0, 5, 24, and 48 h). The concentration of 2.4 mM was selected based on the levels produced by high Try producers *in vitro* (**Figure 1**) and on previous studies evaluating colonic tissues response to Try in Ussing chamber assays (Williams et al., 2014; Bhattarai et al., 2018).

**Figure 3:**
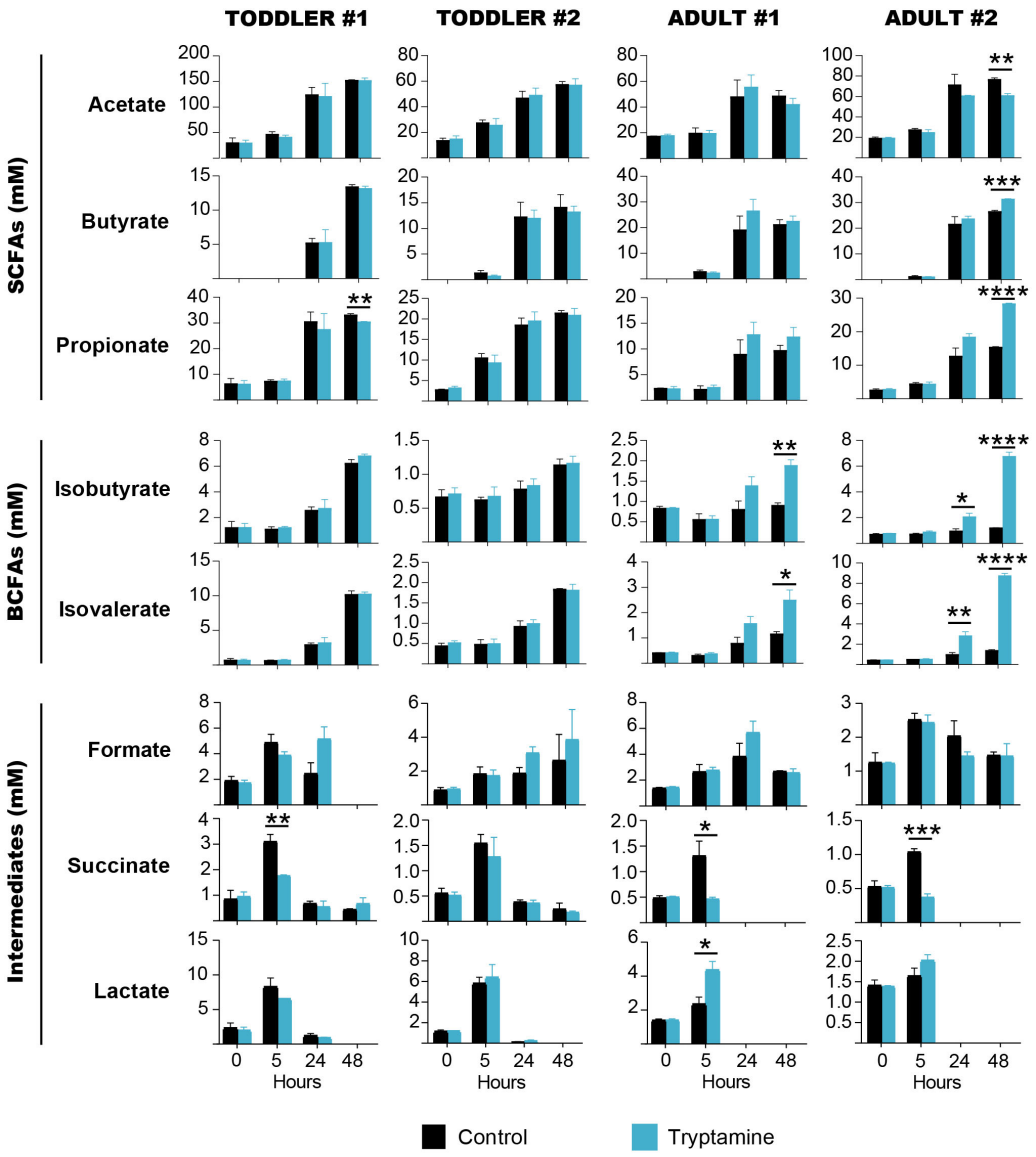
Short-chain fatty acids, branched-chain fatty acids and intermediate metabolites quantified throughout 48 h of batch fermentations of toddler (*n* = 2) or adult (*n* = 2) gut microbiota in the absence (black) or presence (blue) of 2.4 mM tryptamine. Data are mean and standard deviation of three independent replicates. Significances were calculated by using unpaired *t*-test. *p < 0.05; **p < 0.01; ***p < 0.001; ****p < 0.0001.

Overall, Try supplementation induced mild effects on the carbohydrate metabolism of all tested intestinal microbial communities. A minor increase of monosaccharide and disaccharide concentrations was observed in the presence of Try as compared to the control early during the fermentation (5 h) in toddler #1 (7.3 mM vs. 2.9 mM lactose; *p* < 0.05), toddler #2 (0.27 mM vs. 0.11 mM glucose and 0.27 mM vs. 0.04 mM galactose; *p* < 0.05), adult #1 (0.56 mM vs. 0.17 mM glucose and 0.77 mM vs. 0.20 mM galactose; *p* < 0.05), and adult #2 (2.2 mM vs. 1.4 mM; *p* < 0.05) (**Supplementary Figure 3**). Since polysaccharides (i.e., fibers) were supplemented in both toddler-mYCFA and adult-mYCFA, this could suggest an increased release of mono/di-saccharides or, more likely, a reduction in carbohydrate consumption by gut microbes. After 5 h, the pH of the milieu was indeed higher in the presence of Try as compared to the control (except for adult #2; **Supplementary Figure 4**), and there was a significant reduction in the concentration of the intermediate metabolite succinate [1.7 mM vs. 3.1 mM in toddler #1, 0.4 mM vs. 1.3 mM in adult #1, and 0.4 mM vs. 1.0 mM in adult #2 (*p* < 0.05); no difference in toddler #2], but not lactate or formate (**Figure 3**).

After 48 h, the end products from carbohydrate (SCFAs) and protein fermentation (BCFAs) were affected by Try supplementation in a donor-specific manner. In adult #2, the concentrations of both SCFAs and BCFAs were significantly altered, with a decrease of acetate (60.8 mM vs. 76.7 mM, *p* = 0.001) and an increase of butyrate (31.4 mM vs. 26.5 mM, *p* = 0.0002), propionate (27.8 mM vs. 15.1 mM, *p* < 0.0001), isobutyrate (6.7 mM vs. 1.2 mM, *p* < 0.0001), and isovalerate (8.7 mM vs. 1.4 mM, *p* < 0.0001; **Figure 3**). In adult #1, Try supplementation did not alter SCFAs but increased isobutyrate (1.9 mM vs. 0.9 mM, *p* = 0.01) and isovalerate levels (2.5 mM vs. 1.2 mM, *p* = 0.02; **Figure 3**). In toddlers’ microbiota, no significant changes of SCFAs and BCFAs were detected in the presence of Try, except for a small but significant decrease of propionate in toddler #1 (30.4 mM vs. 33.1 mM, *p* = 0.003; **Figure 3**). With all four tested gut microbial communities, no significant reduction of Try concentration was observed throughout the fermentation (**Supplementary Figure 5**), indicating that tryptamine was not metabolized.

We suspected that the observed metabolic changes could be associated with an alteration of the microbial community. Therefore, we performed 16S rRNA metabarcoding for 5- and 48-h fermentation samples from all donors. At the phylum level, Try mediated a significant decrease of the mean relative abundance of the phylum *Bacteroidota* after 5 h in all donors (toddler #1: 25% vs. 32%, *p* = 0.02; toddler #2: 10% vs. 16%, *p* = 0.03; and adult #2: 25% vs. 28%, *p* = 0.02), except for adult #1 where a non-significant reduction was observed (16% vs. 29%, *p* = 0.09; **Supplementary Figure S6A**). A similar pattern was observed at the class level for *Bacteroidia* (**Supplementary Figure 6B**; **Supplementary Table 2**). The reduction of *Bacteroidota* remained significant after 48 h only in toddler #1 (15% vs. 30%, *p* = 0.0003), along with an increase of *Actinobacteriota* (34% vs. 29%, *p* = 0.005) and *Firmicutes* (50% vs. 40%, *p* = 0.0001). A bloom of *Proteobacteria* was observed in adult #2 in the presence of Try (48 h: 5% vs. 2%, *p* = 0.0002; **Supplementary Figure 6A**).

**Figure 4:**
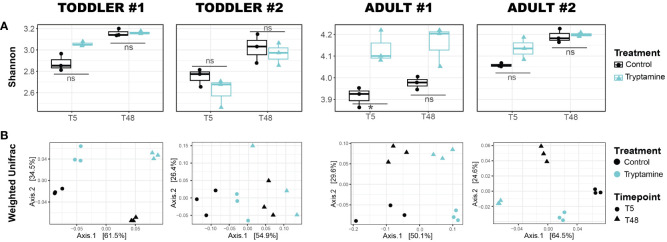
Alteration of toddler and adult gut microbial communities after 5 and 48 h of growth in the absence (control) or presence of 2.4 mM tryptamine. **(A)** Alpha diversity via Shannon index. Significances were calculated by using unpaired *t*-test. *p < 0.05; ns, not significant (*p* > 0.05). **(B)** Beta diversity via weighted UniFrac distance matrix. For each condition, independent replicates are shown. T5, 5-h growth; T48, 48-h growth.

For all four tested microbiota, the observed ASVs (richness; **Supplementary Figure 7A**) and the Shannon index (both richness and evenness; **Figure 4A**) remained stable whether Try was present or absent. However, a small yet significant increase in the Shannon index was noted at 5 h in adult #1 (**Figure 4A**). The beta-diversity analyses showed a distinct clustering of samples treated with Try compared to the control samples (regardless of the time point) when considering the taxa abundance, as indicated by the weighted UniFrac distance matrix (*p* < 0.05 for all donors except adult #1; **Figure 4B**). However, this pattern was not observed when considering the presence or absence of taxa only, as shown by the unweighted UniFrac matrix (all donors non-significant; **Supplementary Figure 7B**). Taken together, our data indicated that Try exerts a mild effect on the overall gut microbiota community structure and on carbohydrate metabolism.

### 3.4 Intestinal bacteria are differently sensitive to tryptamine

Although limited, the donor-specific alterations observed in all four fecal-derived microbiota samples (**Figures 3**, **4**) suggest that certain microbes, rather than the whole community, may be particularly sensitive to the presence of Try. In turn, such specific growth inhibition could alter the microbial community structure and the cross-feeding interactions between microbes. To identify the specific taxa affected by Try, we performed differential abundance analysis at the ASV level (DESeq2 analysis). Considering the two timepoints (5 and 48 h) and the four donors, a total of 145 ASVs were altered by the presence of Try (log2fold change > 1; *p* < 0.05; **Supplementary Figure 8**). One ASV, specifically *Blautia* sp. (ASV005), decreased across all tested conditions. In all adult microbiota samples, *Faecalibacterium* sp. (ASV012), *Blautia massiliensis* (ASV010), and *Blautia faecis* (ASV077) were reduced, and *Coprococcus comes* (ASV024) was increased. Notably, *Bacteroides vulgatus* (ASV001) was decreased in all toddler #1 and adult #2 samples.

**Figure 5:**
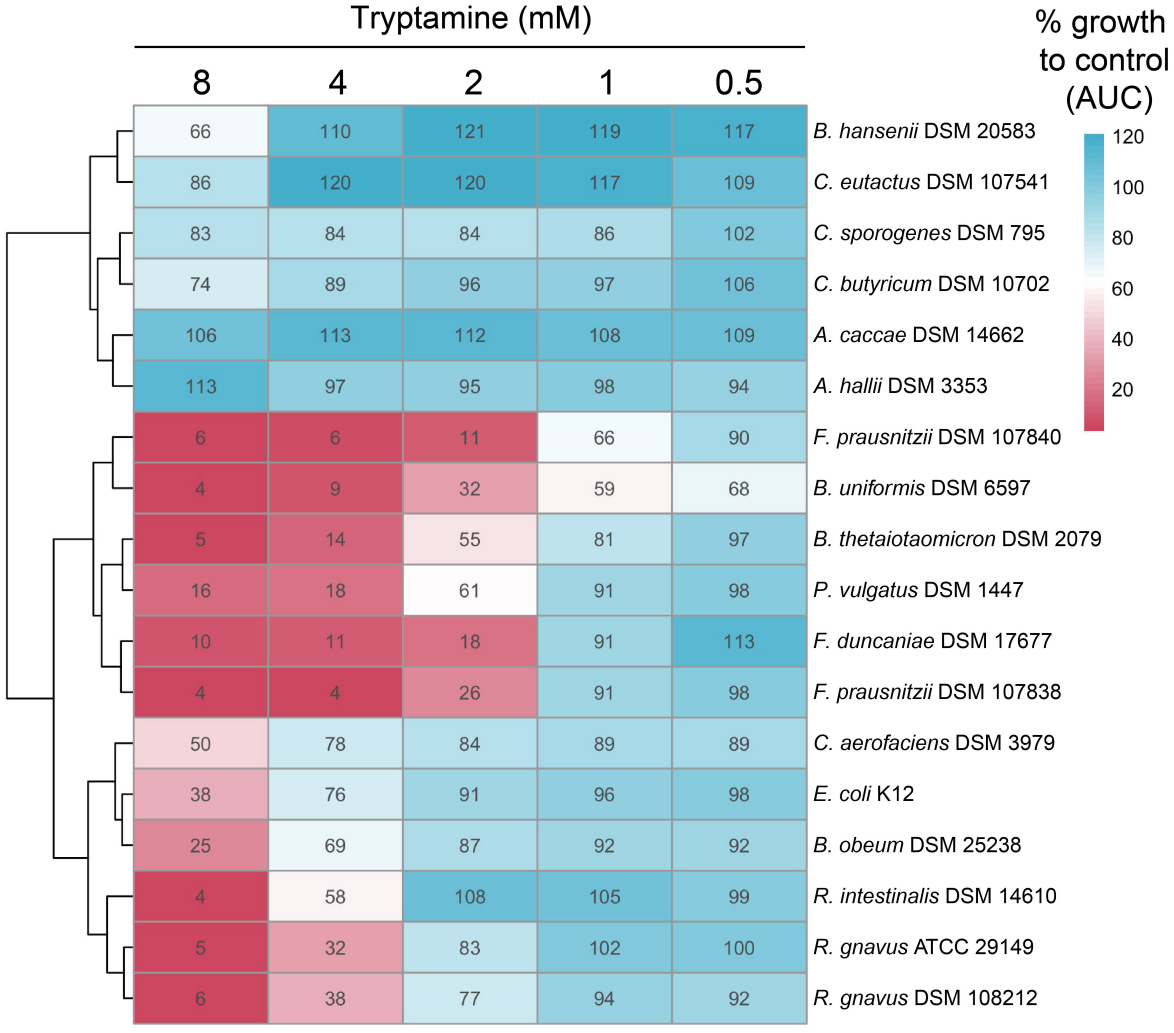
Tryptamine alters the growth of specific gut microbes. Growth of selected intestinal strains exposed to increasing concentrations of tryptamine (from 0.5 to 8 mM) compared to the control (% AUC). The same growth is indicated by 100% as in the control. The data are the mean of two independent replicates.

To determine whether Try possesses inhibitory properties against specific intestinal strains, we cultivated a range of gut bacteria, including some of those identified as downregulated ASVs in the DESeq2 analysis, and exposed them to Try, ranging from 0.5 to 8 mM. This range was chosen to ensure comparability with the 2.4-mM concentration used in *ex vivo* fecal cultivation and included a condition within the micromolar range. Percentage growth as compared to the control without Try was determined using area under the curve (AUC). Overall, the lowest concentration of Try tested (0.5 mM) had little to no effect on the growth of most tested strains, except for *Bacteroides uniformis* DSM 6597, which only grew to 68% of the control (**Figure 5**). At 2 mM Try, all three *Bacteroides* strains tested were affected, with 61% (*Phocaelicola vulgatus* DSM 1447, previously *B. vulgatus*), 55% (*B. thetaiotaomicron* DSM 2079), and 32% (*B. uniformis* DSM 6597) growth compared to the control (**Figure 5**). Exposure to 2 mM Try also significantly inhibited the growth of the three *Faecalibacterium* strains tested, with 26% (*F. prausnitzii* DSM 107838), 18% (*F. duncaniae* DSM 17677), and 11% (*F. prausniitzii* DSM 107840) growth compared to the control (**Figure 5**). *Blautia obeum* DSM 25238 was not strongly affected by Try, with 69% growth observed at 4 mM (**Figure 5**). Interestingly, the growth of the Try producers *R. gnavus* ATCC 29149 and DSM 108212 was reduced at 4 mM, with 32% and 38% growth compared to the control, respectively, which was not the case for *C. sporogenes* DSM 795 and *B. hansenii* DSM 20583 (**Figure 5**). Several other strains, including *Coprococcus eutactus* DSM 107541, *Anaerostipes caccae* DSM 14662, and *Anaerobutyricum hallii* DSM 3353, were not inhibited by Try at any concentration (**Figure 5**). Interestingly, upon reinoculation (1%, v/v) into Try-free fresh medium, most strains recovered from exposure to Try concentrations of 4 mM and below, except for the *Faecalibacterium* strains (**Supplementary Figure 9**). Collectively, our data suggest that Try exhibits inhibitory properties, diminishing the growth of specific intestinal bacteria while others remain unaffected.

## 4 Discussion

Tryptamine acts as a neuromodulator in the human body (Borowsky et al., 2001; Zucchi et al., 2006; Fontanilla et al., 2009); however, little is known about its functionality for microbes. Within the gut, Try levels have been shown to be greatly reduced in germ-free animals (Marcobal et al., 2013), indicating that its production is mediated by the gut microbiota. It is estimated that 10% of the human population harbors gut bacteria that can produce Try through the process of Trp decarboxylation (Williams et al., 2014). There are, however, very few studies that have evaluated and characterized Try production by gut bacteria (Roager and Licht, 2018). Tryptamine production has been first identified in two intestinal strains *in vitro*, i.e., *R. gnavus* ATCC 29149 and *C. sporogenes* ATCC 15579, with a homology analysis suggesting that other taxa from the Lachnospiraceae family may also produce it (Williams et al., 2014). Here we confirmed the production in five new strains, namely, *C. nexile* DSM 1787, *E. asparagiformis* DSM 15981, *B. hansenii* DSM 20583, *R. gnavus* DSM 108212, and *C. sporogenes* DSM 795, as recently reported (Sugiyama et al., 2022). Under the tested *in vitro* conditions, the strains produced Try at levels ranging from 16 to 3,383 μM after 48 h, which is similar to the levels previously reported in *R. gnavus* ATCC 29149 (~1.7 mM after 72 h) (Williams et al., 2014). Note that we cannot exclude the possibility that the tested non-producing strains may be capable of production under different conditions.

Tryptamine production was mixed-growth-associated, starting during the exponential phase and continuing during the stationary phase (Sakthiselvan et al., 2019). This pattern suggests that tryptophan decarboxylase activity may not be regulated and induced in response to physiological stressors (e.g., nutrient depletion or pH changes) as previously demonstrated for other biogenic amines (Bargossi et al., 2015; Barbieri et al., 2019; Otaru et al., 2021). In line with this, our results indicate that Trp decarboxylation to Try was not promoted at low pH under the tested condition and may not protect the cells of *R. gnavus* DSM 108212 and *C. sporogenes* DSM 795 against acid stress, unlike other known amino acid-dependent acid tolerance systems (e.g., lysine, arginine, or glutamate decarboxylase systems) (Guan and Liu, 2020). Further research is needed to elucidate the precise regulation, function, and benefits of Try production for intestinal bacterial cells.

Although results may substantially differ in an *in vivo* environment, the *ex vivo* data obtained from exposing complex gut microbial communities to 2.4 mM Try might provide preliminary insights into some of Try’s properties, particularly its ability to mediate physiological changes in certain gut microbes. All four fecal-derived microbiota tested displayed an altered metabolism in response to Try, with a decrease of carbohydrate consumption early during batch fermentation experiments, yet the consequences on the end metabolites SCFAs and BCFAs were donor specific. With one microbiota (adult #2), an increase of butyrate was observed, in line with an animal study showing that an intraperitoneal injection of Try could alter the gut microbiota and increase the butyrate cecal levels (Dopkins et al., 2021). The BCFAs were increased in both adult donors, suggesting a change of microbial communities and/or metabolism that could favor protein fermentation (Diether and Willing, 2019).

At the taxonomic level, we showed that Try exerted a mild effect on the structure of the gut microbiota community, leading to changes in the abundance of specific taxa (weighted UniFrac distance) rather than their presence or absence (unweighted UniFrac distance). Particularly, the phylum *Bacteroidota*, along with several species from the genus *Bacteroides*, was significantly decreased in response to Try, similar to what was previously reported in mice treated with Try (Dopkins et al., 2021). Using pure cultures, we confirmed the significant growth reduction at 2 mM Try for all the three *Bacteroides* species tested. *Bacteroides* are primary degraders of fiber and produce mainly succinate in the presence of high CO_2_ concentration (Fischbach and Sonnenburg, 2011) (*ex vivo* fermentations conducted with 100% CO_2_ in the headspace). Their inhibition could, at least in part, explain the reduced production of succinate observed with three donor microbiota early (5 h) during fermentation. Since *Bacteroides* spp. and the Try producer *R. gnavus* are both mucin-degrading taxa residing in the mucus layer (Glover et al., 2022), the secretion of Try by *R. gnavus* might provide advantage over *Bacteroide*s spp. for the colonization of this intestinal niche. *Faecalibacterium* was also affected by Try, as shown during *ex vivo* batch fermentation with both adults’ microbiota and subsequently confirmed *in vitro* using pure cultures from three strains. *Faecalibacterium* spp. are butyrate producers known to be highly sensitive to oxygen (Duncan et al., 2002); however, their sensitivity to Try has not been described. Despite their decreased abundance, the butyrate levels increased after 48 h of fermentation with adult #2 microbiota, potentially due to the observable increase of other butyrate-producing taxa, i.e., *Agathobacter*, *Anaerostipes*, *Coprococcus*, and *Roseburia* (Amiri et al., 2022). When tested in pure culture, the butyrate-producing strains *A. caccae* DSM 14662, *A. hallii* DSM 3353, *C. butyricum* DSM 10702, *C. eutactus* DSM107541, and *R. intestinalis* DSM 14610 were slightly or not affected by Try.

To date, there are very few studies that have looked at the inhibitory properties of Try in bacteria. One study using growth supernatants from soil *Clostridium* spp. suggested that Try could be one of the active antimicrobial agents inhibiting *Bacillus mycoides* ATCC 6462, *Bacillus cereus* NZRM 5, and *Pseudomonas aeruginosa* ATCC 25668 (Pahalagedara et al., 2020). Another study showed that Try reduced the aerobic growth of *E. coli* K12 (at >0.6 mM), *Serratia marcescens* DSM 48 (at >0.75 mM), and *P. aeruginosa* PAO1 (at >1.5 mM) (Luqman et al., 2021). In our work, *E. coli* K12 was not strongly affected by up to 4 mM Try under anaerobic conditions, and the difference observed may be explained by the presence/absence of oxygen. Overall, the mechanisms behind the bacterial inhibition by Try are unknown. In eukaryotic cells, it has been suggested that Try toxicity arises from its competitive inhibition with Trp for the enzyme tryptophanyl-rRNA synthetase, which is vital for protein biosynthesis (Paley, 1999). Further research is necessary to elucidate the specific mechanisms of Try toxicity in bacteria.

## 5 Conclusions

In conclusion, we identified intestinal strains capable of transforming Trp into Try. We showed that Try formation was mixed-growth-associated and was not promoted by low pH. Further research is needed to understand the regulatory mechanisms of Try in bacteria. In turn, secreted Try can influence the gut microbial community *ex vivo*, inducing mild metabolic and taxonomic changes. Specifically, we showed that the growth of *Bacteroides* and *Faecalibacterium* was reduced in the presence of Try. Additional studies are necessary to clarify the inhibitory mechanisms of Try toward specific strains while leaving others unaffected.

## Data availability statement

The datasets presented in this study can be found in online repositories. The names of the repository/repositories and accession number(s) can be found below: http://www.ncbi.nlm.nih.gov/bioproject/1063610, NCBI BioProject PRJNA1063610.

## Ethics statement

The requirement of ethical approval was waived by the ethics committee of ETH Zürich for the studies involving humans because the fecal sample collection procedure was not performed under conditions of intervention, and it was carried out in an anonymized manner. The studies were conducted in accordance with the local legislation and institutional requirements. Written informed consent for participation in this study was provided by the participants’ legal guardians/next of kin.

## Author contributions

NO: Conceptualization, Data curation, Formal analysis, Investigation, Methodology, Project administration, Supervision, Writing – review & editing. AG: Data curation, Formal analysis, Software, Visualization, Writing – original draft, Writing – review & editing. SP: Data curation, Investigation, Methodology, Writing – review & editing. JZ: Data curation, Formal analysis, Investigation, Methodology, Software, Visualization, Writing – review & editing. DM: Data curation, Investigation, Methodology, Writing – review & editing. JB: Data curation, Formal analysis, Investigation, Methodology, Writing – review & editing. EK: Data curation, Investigation, Methodology, Writing – review & editing. CL: Funding acquisition, Investigation, Resources, Supervision, Validation, Writing – review & editing. BP: Conceptualization, Data curation, Formal analysis, Funding acquisition, Investigation, Methodology, Project administration, Resources, Supervision, Validation, Visualization, Writing – original draft, Writing – review & editing.
