# Supplementary material for: Intestinal bacteria-derived tryptamine and its impact on human gut microbiota
Source: Front Microbiomes. 2024 Apr 3;3:1373335. doi: 10.3389/frmbi.2024.1373335 (PMC12993505; doi:10.3389/frmbi.2024.1373335)
Supplement: Supplementary file 1 [file DataSheet_1.pdf]

SUPPLEMENTARY TABLES AND FIGURES

Table S1. Bacterial strains used in this study, for the screening of tryptamine-producers and/or to evaluate tryptamine sensitivity in individual strains.

Genus	Species	Strain	Strain used for:		Isolation source	Culture collection
			Screening	Sensitivity		
Anaerobutyricum	*hallii*	DSM 3353		X	human feces	DSMZ
Anaerostipes	*caccae*	DSM 14662		X	human feces	DSMZ
Bacteroides	*thetaiotaomicron*	DSM 2079		X	human feces	DSMZ
Bacteroides	*uniformis*	DSM 6597		X	human feces	DSMZ
Blautia	*obeum*	DSM 25238	X	X	human feces	DSMZ
Blautia	*hydrogenotrophica*	DSM 10507	X		human feces	DSMZ
Blautia	*producta*	DSM 14466	X		human feces	DSMZ
Blautia	*hansenii*	DSM 20583	X	X	human feces	DSMZ
Clostridium	*nexile*	DSM 1787	X		human feces	DSMZ
Clostridium	*sporogenes*	DSM 795	X	X	soil	DSMZ
Clostridium	*butyricum*	DSM 10702		X	pig intestine	DSMZ
Collinsella	*aerofaciens*	DSM 3979		X	human feces	DSMZ
Coprococcus	*eutactus*	DSM 107541		X	human feces	DSMZ
Enterocloster	*asparagiformis*	DSM 15981	X		human feces	DSMZ
Escherichia	*coli*	K12		X	human feces	FBT
Faecalibacterium	*duncaniae*	DSM 17677		X	human feces	DSMZ
Faecalibacterium	*prausnitzii*	DSM 107838		X	human feces	DSMZ
Faecalibacterium	*prausnitzii*	DSM 107840		X	human feces	DSMZ
Mediterraneibacter	*glycyrrhizinilyticus*	DSM 17593	X		human feces	DSMZ
Phocaeicola	*vulgatus*	DSM 1447		X	human feces	DSMZ
Roseburia	*intestinalis*	DSM 14610		X	human feces	DSMZ
Ruminococcus	*bromii*	ATCC 27255	X		human feces	ATCC
Ruminococcus	*gauvreauii*	DSM 19829	X		human feces	DSMZ
Ruminococcus	*gnavus*	ATCC 29149	X	X	human feces	ATCC
Ruminococcus	*gnavus*	DSM 108212	X	X	human feces	DSMZ
"Lachnospiraceae bacterium"		DSM 24404	X		human gut biopsy	DSMZ

DSMZ: German Collection of Microorganisms and Cell Culture GmbH; ATCC: American Type Culture Collection; FBT: Laboratory of Food Biotechnology, ETH Zürich.

Table S2. Taxonomic relative abundance of toddler and adult microbial communities at the class level after 5 and 48 h fermentation in absence (control) or presence of 2.4 mM tryptamine. Mean values of the relative abundance (%) are provided, along with the adjusted P value (multiple unpaired t-tests, corrected for multiple comparisons using the Holm-Šídák method).

	**5 h**			**48 h**		
**Toddler #1**	**Control (mean %)**	**Tryptamine (mean %)**	**Adjusted P Value**	**Control (mean %)**	**Tryptamine (mean %)**	**Adjusted P Value**
Bacteroidia	32,46	25,26	0,0264	30,21	15,22	0,0001
Actinobacteria	22,69	27,68	0,0549	22,1	27,98	0,0073
Negativicutes	3,336	5,537	0,0243	15,92	18,7	0,0228
Coriobacteriia	7,375	6,657	0,1834	7,036	5,742	0,0539
Clostridia	25,53	18,35	0,0052	17,58	20,06	0,0551
Bacilli	6,381	12,5	0,0008	6,286	11,17	0,0003
γ-proteobacteria	1,806	3,029	0,0837	0,7572	0,9307	0,6388
Verrucomicrobiae	0,3707	0,915	0,0359	0,09465	0,2051	0,2163
Desulfovibrionia	0	0,007888	0,4929	0	0	
Lentisphaeria	0	0		0	0	
Vampirivibrionia	0	0		0,007888	0	0,6388
Methanobacteria	0	0		0,007888	0	0,6388
α-proteobacteria	0	0		0	0	
Fusobacteriia	0,04733	0,07099	0,4950	0	0	
	**5 h**			**48 h**		
**Toddler #2**	**Control (mean %)**	**Tryptamine (mean %)**	**Adjusted P Value**	**Control (mean %)**	**Tryptamine (mean %)**	**Adjusted P Value**
Bacteroidia	15,81	10,18	0,0440	10,85	7,911	0,5673
Actinobacteria	13,98	17,82	0,1286	15,63	18,48	0,5673
Negativicutes	40,05	46,87	0,2025	47,54	45,26	0,9842
Coriobacteriia	2,453	3,037	0,3594	1,215	1,238	0,9842
Clostridia	18,12	11,41	0,3594	12,1	10,1	0,6647
Bacilli	2,169	2,627	0,4750	5,506	8,732	0,1874
γ-proteobacteria	7,367	8,03	0,8866	7,107	8,235	0,9842
Verrucomicrobiae	0,04733	0,03155	0,8866	0,05521	0,04733	0,9842
Desulfovibrionia	0	0		0	0	
Lentisphaeria	0	0		0	0	
Vampirivibrionia	0	0		0	0	
Methanobacteria	0	0		0	0	
α-proteobacteria	0	0		0	0	
Fusobacteriia	0	0		0	0	
	**5 h**			**48 h**		
**Adult #1**	**Control (mean %)**	**Tryptamine (mean %)**	**Adjusted P Value**	**Control (mean %)**	**Tryptamine (mean %)**	**Adjusted P Value**
Bacteroidia	29,31	16,45	0,1303	23,37	16,3	0,1769
Actinobacteria	2,713	6,089	0,1836	3,021	4,733	0,2609

Negativicutes	0,9465	0,773	0,9430	3,455	2,989	0,9016
Coriobacteriia	0,01578	0,03155	0,9426	0,07888	0,1341	0,8741
Clostridia	61,21	66,49	0,2917	65,91	71,6	0,6178
Bacilli	4,133	8,613	0,1836	1,57	1,861	0,9381
γ-proteobacteria	1,294	1,152	0,9430	2,437	2,272	0,9381
Verrucomicrobiae	0,2603	0,1972	0,9430	0,07099	0,03155	0,6178
Desulfovibrionia	0,1104	0,1183	0,9430	0,05521	0,0631	0,9381
Lentisphaeria	0	0,007888	0,8696	0	0	
Vampirivibrionia	0,007888	0,07099	0,6456	0,03155	0,01578	0,9381
Methanobacteria	0	0		0	0	
α-proteobacteria	0	0		0	0	
Fusobacteriia	0	0		0	0	
	**5 h**			**48 h**		
**Adult #2**	**Control (mean %)**	**Tryptamine (mean %)**	**Adjusted P Value**	**Control (mean %)**	**Tryptamine (mean %)**	**Adjusted P Value**
Bacteroidia	28,04	24,64	0,0445	21,15	18,91	0,3271
Actinobacteria	5,151	2,698	0,0029	3,676	1,877	0,0322
Negativicutes	2,059	3,92	0,0003	4,733	3,699	0,2495
Coriobacteriia	3,51	5,206	0,1306	6,783	10,39	0,0106
Clostridia	58,98	59,45	0,8875	60,13	57	0,0612
Bacilli	1,254	2,997	0,0445	1,238	2,603	0,0051
γ-proteobacteria	0,5679	0,5364	0,8875	2,122	5,34	0,0009
Verrucomicrobiae	0,3313	0,4102	0,8305	0,1183	0,07888	0,6388
Desulfovibrionia	0,03155	0,1025	0,5518	0,01578	0,0631	0,1687
Lentisphaeria	0,07099	0,02366	0,4728	0,02366	0,03155	0,6388
Vampirivibrionia	0	0		0	0	
Methanobacteria	0,007888	0	0,7428	0	0	
α-proteobacteria	0	0,01578	0,4005	0,007888	0	0,6388
Fusobacteriia	0	0		0	0	

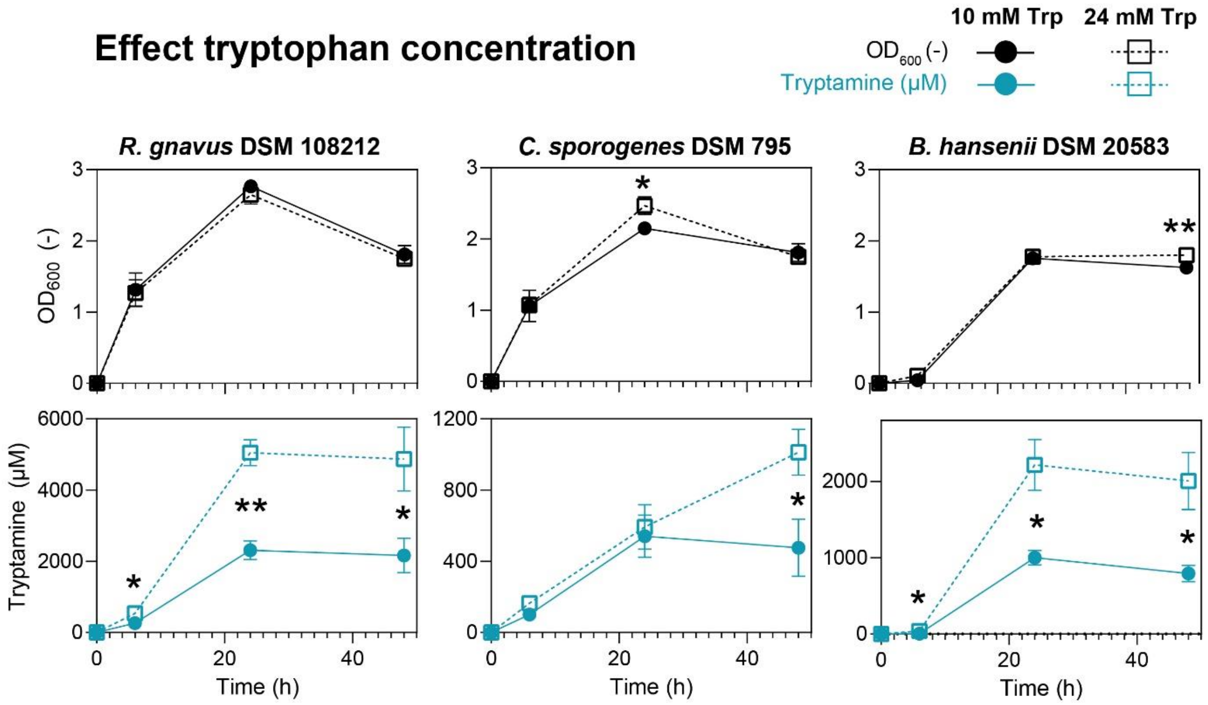

Figure S1: Effect of initial tryptophan (Trp) concentration on the growth (OD_{600}) and tryptamine production in *R. gnavus* DSM 108212, *C. sporogenes* DSM 795, and *B. hansenii* DSM 20583. Strains were grown in mYCFA containing 10 or 24 mM Trp (pH 6.5). All strains were incubated at 37°C for 48 h. Data are mean and standard deviation of three independent replicates. Significances were calculated by unpaired t-test. * $p < 0.05$; ** $p < 0.01$; *** $p < 0.001$; **** $p < 0.0001$.

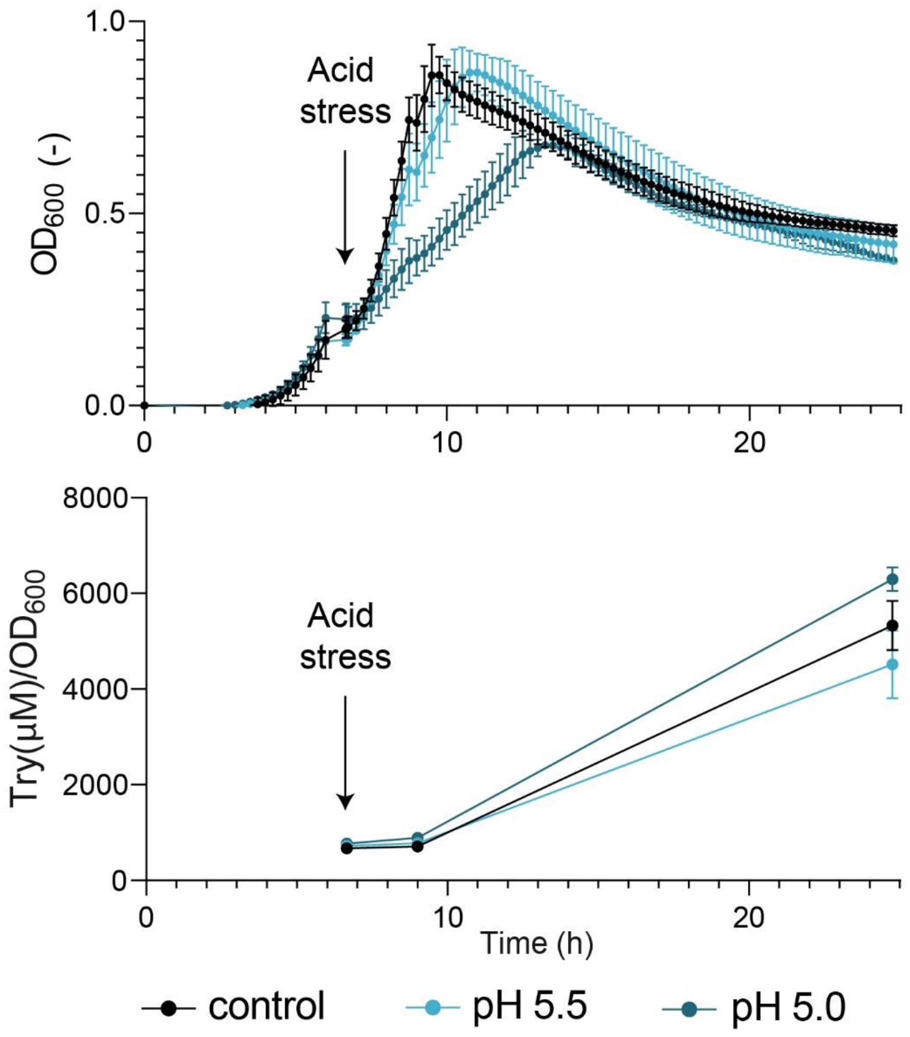

Figure S2. **Growth (OD_{600}) and tryptamine (Try) production (normalized by OD_{600}) in response to acid stress applied to actively growing cells of *R. gnavus* ATCC 29149**. Cells were incubated at 37°C for 24 h in mYCFA containing 5 mM Trp. When indicated (OD_{600} ~0.2), pH was reduced (from 6.2; control) to 5.5 and 5.0 using anaerobic 2.5 M HCl. Try was quantified 2 and 18 h post-stress. Data are mean and standard deviation of three independent replicates. Significances were calculated by unpaired t-test.

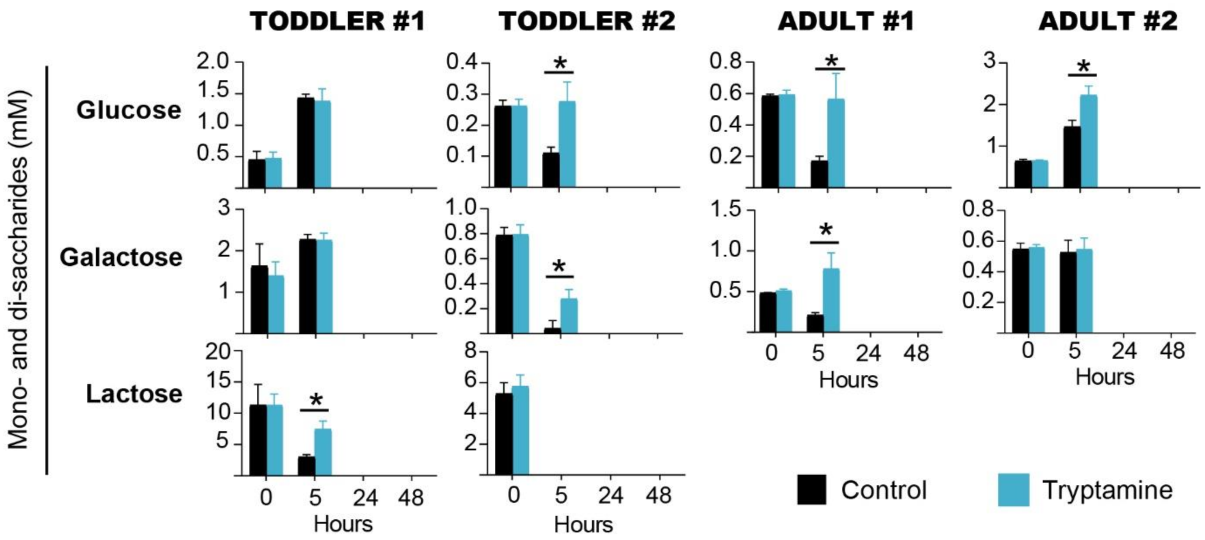

Figure S3. Mono- and di-saccharides quantified throughout 48 h batch fermentations of toddler (n=2) or adult (n=2) gut microbiota, in absence (black) or presence (blue) of 2.4 mM tryptamine. Data are mean and standard deviation of three independent replicates. Significances were calculated by unpaired t-test. * $p < 0.05$.

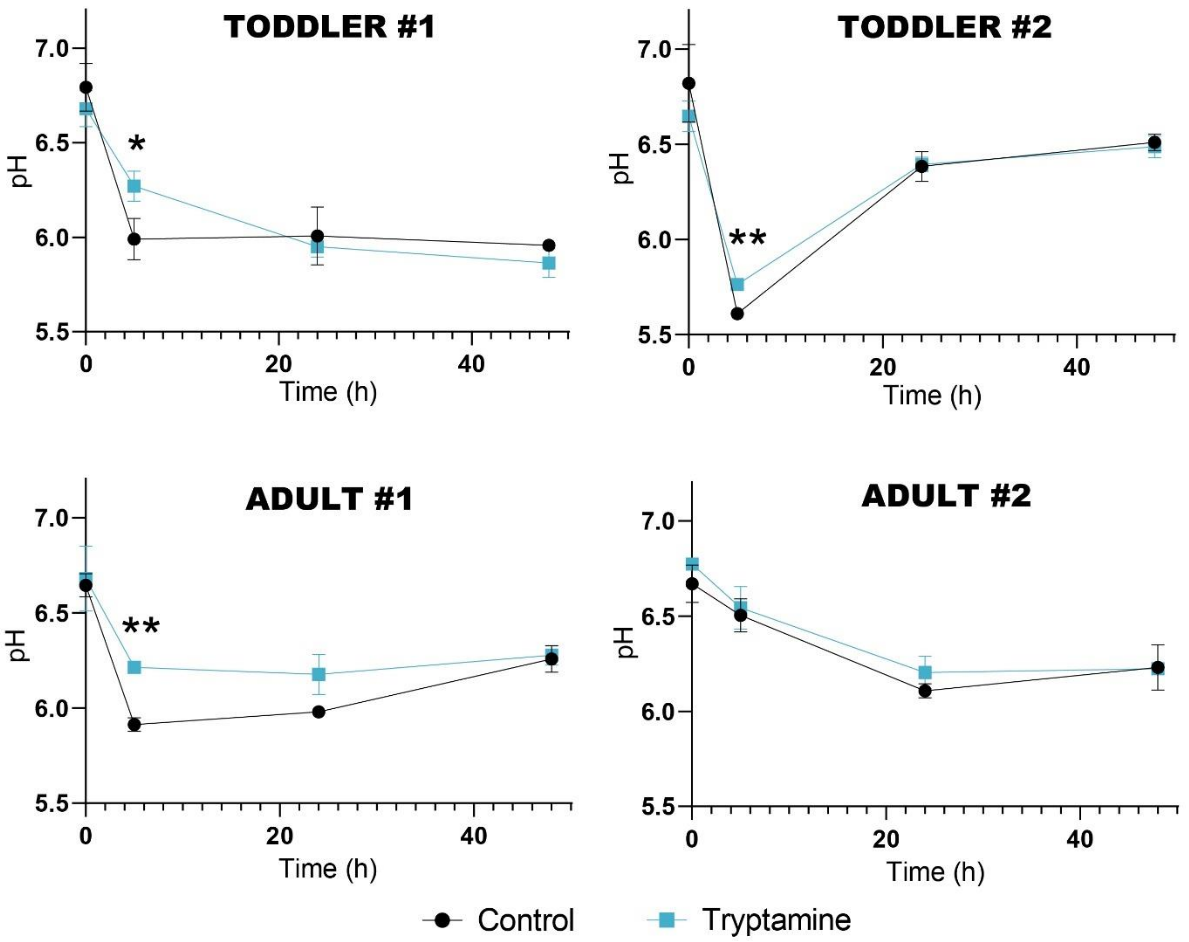

Figure S4. pH profiles during 48 h anaerobic batch fermentations of toddler (n=2) or adult (n=2) fecal microbiota, in absence (black) or presence (blue) of 2.4 mM tryptamine. Data are mean and standard deviation of three independent replicates. Significance was calculated by unpaired t-test. * $p < 0.05$**; $p < 0.01$.

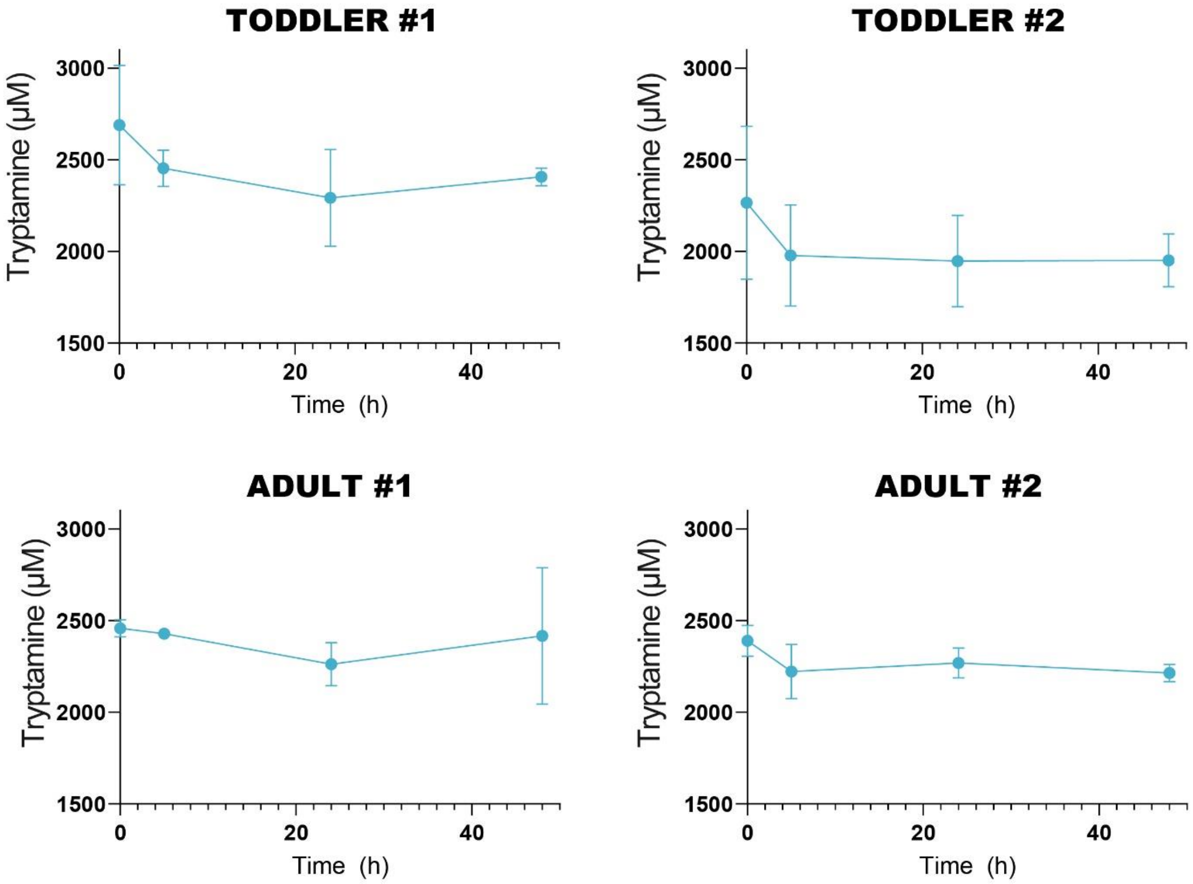

Figure S5. Tryptamine concentration throughout 48 h anaerobic batch fermentations of toddler (n=2) or adult (n=2) fecal microbiota in presence of 2.4 mM tryptamine. Data are mean and standard deviation of three independent measurements.

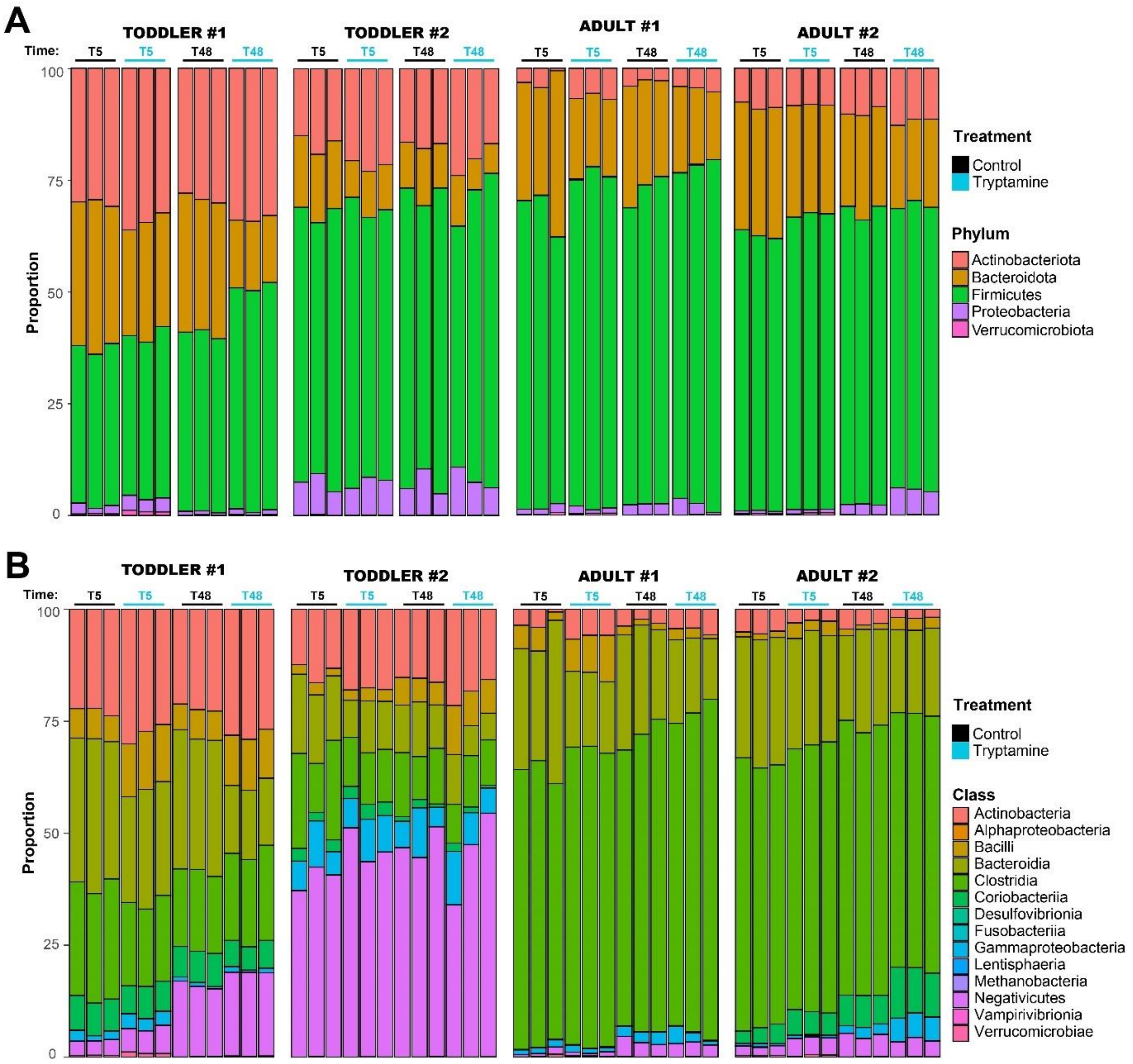

Figure S6. Taxonomic relative abundance of toddler and adult microbial communities, after 5 and 48 h fermentation in absence (control) or presence of 2.4 mM tryptamine. A) Phylum level. B) Class level. For each condition, independent replicates are shown. T5: 5 h growth; T48: 48 h growth.

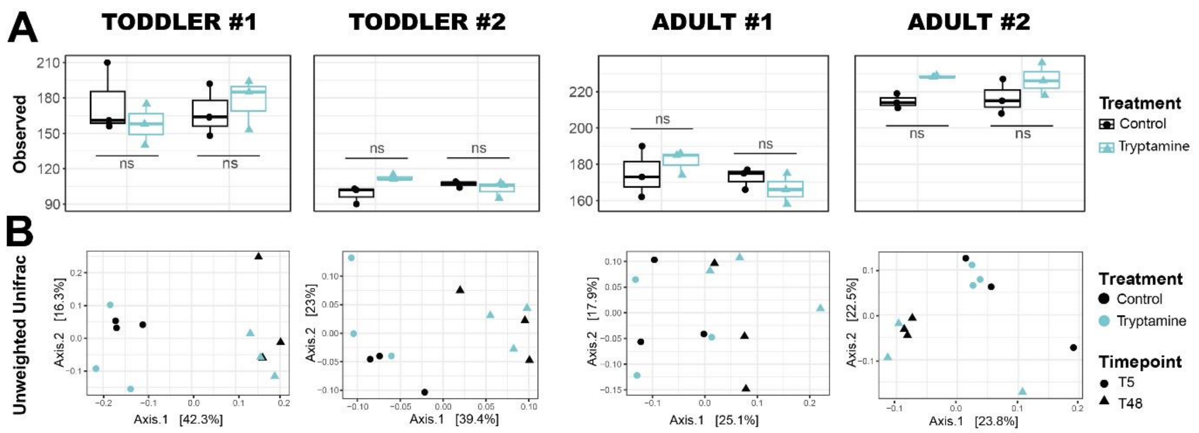

Figure S7. **Alteration of toddler and adult gut microbial communities after 5 and 48 h growth in absence (control) or presence of 2.4 mM tryptamine**. A) Alpha-diversity *via* Observed species. Significances were calculated by unpaired t-test. ns: not significant ($p > 0.05$). B) Beta-diversity *via* unweighted Unifrac distance matrix. For each condition, independent replicates are shown. T5: 5 h growth; T48: 48 h growth.

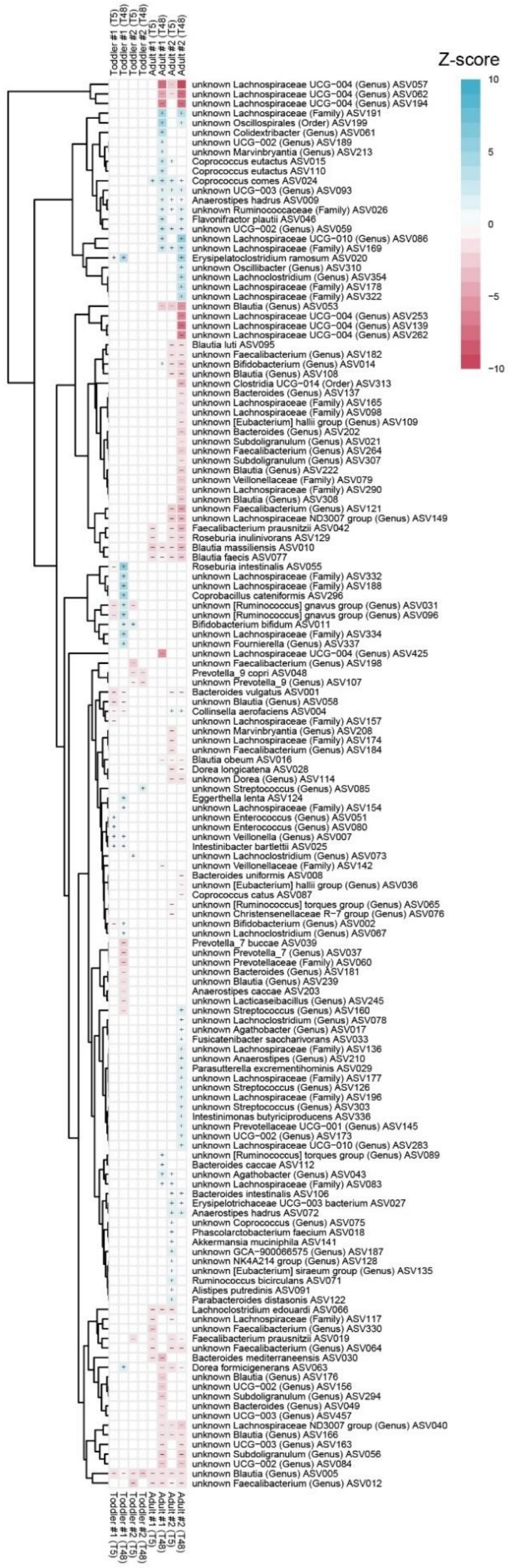

Figure S8. **Tryptamine alters the growth of specific gut microbes.** Significantly different abundance (scaled to z-score: considering Log2Fold change>1; p<0.05) of taxa in cultured microbiota from toddlers (#1 and #2) and adults (#1 and #2) after 5 (T5) and 48 h (T48) growth in presence of 2.4 mM tryptamine. Per each ASV, red indicates significantly lower abundance, while blue indicates significantly higher abundance between control and tryptamine treatment. Data are mean of three independent replicates.

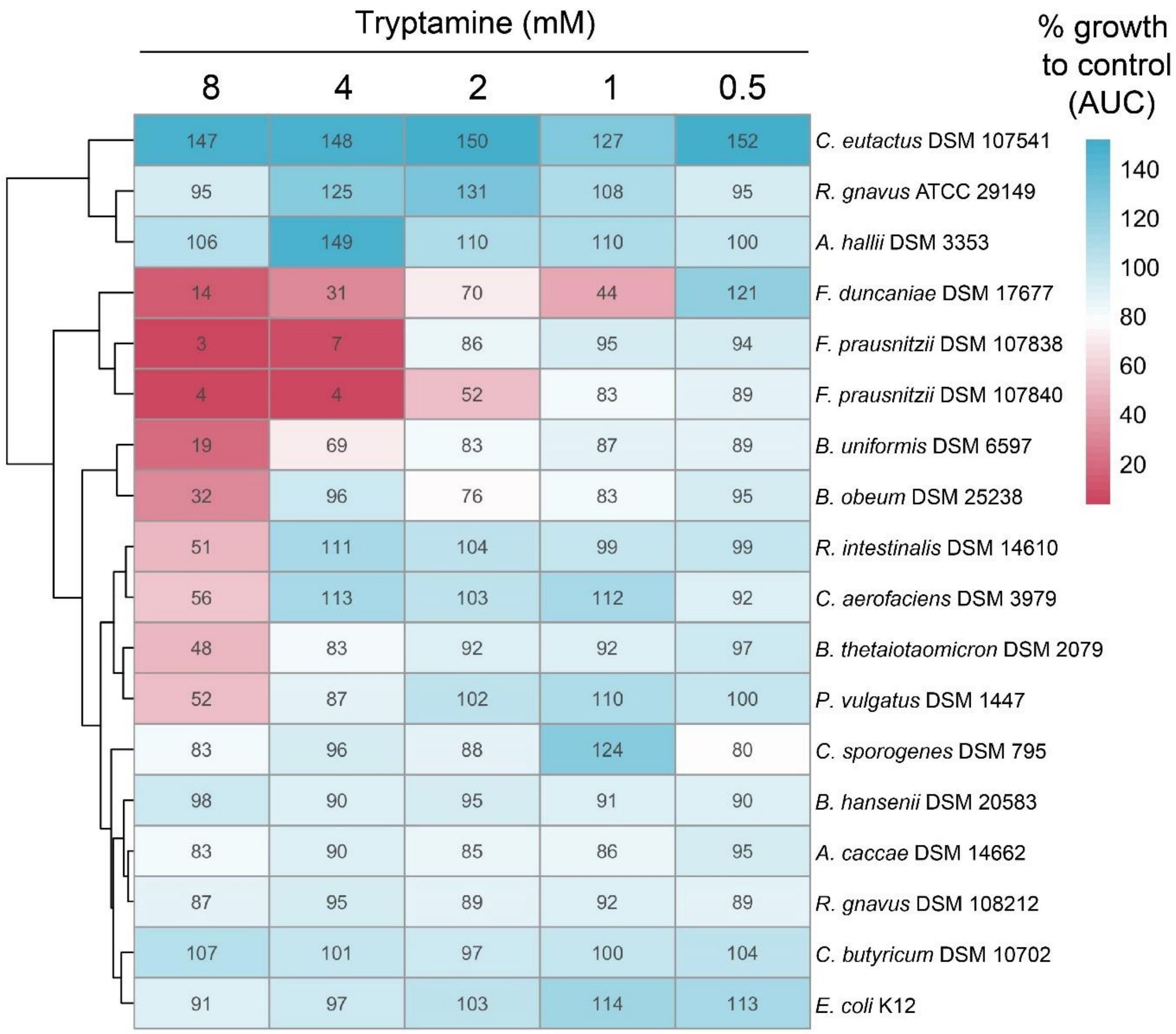

Figure S9. **Effect of tryptamine exposure on the growth of specific gut microbes after re-inoculation in tryptamine-free media.** Strains previously exposed to tryptamine (Figure 5; from 0.5 to 8 mM) were regrown in fresh mYCFA medium without tryptamine. 100% indicates the same growth as in the control (previously exposed to tryptamine-free mYCFA). Data are mean of two independent replicates.
